# Sin máscara: las condiciones laborales en las plataformas digitales de trabajo durante la pandemia de COVID‐19

**DOI:** 10.1111/ilrs.12249

**Published:** 2022-09-06

**Authors:** Kelle HOWSON, Funda USTEK‐SPILDA, Alessio BERTOLINI, Richard HEEKS, Fabian FERRARI, Srujana KATTA, Matthew COLE, Pablo AGUERA RENESES, Nancy SALEM, David SUTCLIFFE, Shelly STEWARD, Mark GRAHAM

**Affiliations:** ^1^ Oxford Internet Institute, Universidad de Oxford; ^2^ Global Development Institute Universidad de Manchester

**Keywords:** economía de plataformas, COVID‐19, futuro del trabajo, plataformas laborales digitales, derechos en el trabajo, empleo precario, condiciones de trabajo

## Abstract

Las plataformas digitales de trabajo se promueven a gran escala como solución a la crisis de desempleo provocada por la pandemia de COVID‐19. Sin embargo, la pandemia también ha puesto de manifiesto la vulnerabilidad de quienes trabajan en ellas en tareas consideradas esenciales. Se examinan aquí las políticas de COVID‐19 de 191 plataformas en 43 países para entender cómo la crisis ha cambiado las convenciones de la economía de plataformas. Utilizando una tipología de «trabajo de plataforma justo», se identifican avances en la protección de las y los trabajadores, pero también problemas significativos, como el afianzamiento del trabajo precario a medida que las plataformas aprovechan las oportunidades derivadas de la crisis.

* Oxford Internet Institute, Universidad de Oxford; kelle.howson@oii.ox.ac.uk (autora para la correspondencia), funda.ustek@oii.ox.ac.uk, alessio.bertolini@oii.ox.ac.uk, fabian.ferrari@oii.ox.ac.uk, srujana.katta@oii.ox.ac.uk, matthew.cole@oii.ox.ac.uk, pablo.aguerareneses@oii.ox.ac.uk, nancy.salem@oii.ox.ac.uk, david.sutcliffe@oii.ox.ac.uk, shelly.steward@oii.ox.ac.uk y mark.graham@oii.ox.ac.uk. ** Global Development Institute, Universidad de Manchester; richard.heeks@manchester.ac.uk. Este trabajo ha contado con financiación de los siguientes organismos: Ministerio Federal de Cooperación Económica y Desarrollo (BMZ) de Alemania, en el marco de un encargo de la Deutsche Gesellschaft für Internationale Zusammenarbeit; Economic and Social Research Council (ESRC, Reino Unido), a través del Global Challenges Research Fund (ES/S00081X/1); Higher Education Innovation Fund y ESRC Impact Acceleration Account, a través del COVID-19: Economic, Social, Cultural, & Environmental Impacts – Urgent Response Fund de la Universidad de Oxford; y Consejo Europeo de Investigación en el marco del programa de financiación de la investigación y la innovación Horizonte 2020 de la Unión Europea (acuerdo de subvención núm. 838081).

La responsabilidad de las opiniones expresadas en los artículos solo incumbe a sus autores, y su publicación en la *Revista Internacional del Trabajo* no significa que la OIT las suscriba.

Artículo original: «Stripping back the mask: Working conditions on digital labour platforms during the COVID-19 pandemic», *International Labour Review* 161 (3). Traducción del equipo editorial de la Revista. Traducido también al francés en *Revue internationale du Travail* 161 (3).

### 1. Introducción

Millones de trabajadoras y trabajadores de todo el mundo dependen cada vez más de la economía de plataformas, al realizar tareas a destajo o puntuales para clientes a través de aplicaciones o plataformas digitales de trabajo. Antes de la pandemia de COVID-19, las investigaciones hacían sonar cada vez más la alarma sobre las condiciones inseguras y precarias a las que se enfrentan las y los trabajadores de plataformas a nivel mundial, temiendo una competencia a la baja a lo que a condiciones de trabajo se refiere en un contexto de vacío normativo (Berg et al. 2019; Forde et al. 2017; Graham y Anwar 2019). Se señaló sobre todo la rápida «plataformización» de diversos tipos de trabajo y el hecho de que este proceso estaba «atrayendo» a más y más trabajadoras y trabajadores fuera del ámbito de las salvaguardias laborales que tanto había costado conseguir y tanto tiempo requería adaptar (Stanford 2017). Cuestiones como la tipificación de las y los trabajadores de plataformas como contratistas independientes o trabajadores autónomos (y su consiguiente exclusión de derechos y prestaciones clave) ya eran muy polémicas entre las partes interesadas en la economía de plataformas anterior a la pandemia. Sin embargo, pocos imaginaban que todas las consecuencias materiales de estos debates se manifestarían de forma tan repentina y devastadora cuando comenzaron los confinamientos y estas trabajadoras y trabajadores se encontraron, en su mayoría, sin poder recurrir a las redes de seguridad social en medio de la crisis económica concomitante. Antes de la pandemia, las personas con mayores capacidades podrían haber pasado por alto el hecho de que el trabajo en plataformas no les proporcionara una red de seguridad, pero los acontecimientos de 2020 pusieron al descubierto hasta qué punto el modelo de plataforma digital de trabajo ha conseguido transferir los riesgos a las trabajadoras y trabajadores, así como las consecuencias reales de este cambio. Estos sucesos también hicieron evidente que las plataformas digitales intermediaban en la realización de tareas esenciales (compras, reparto, transportes, trabajo de cuidados) para mantener no solo nuestras economías, sino incluso la salud de nuestras comunidades, especialmente en tiempos de crisis de salud pública.

Desde el comienzo de la pandemia existe una necesidad imperiosa de datos y conocimientos sobre cuántas trabajadoras y trabajadores dependen de las plataformas digitales de trabajo para su sustento, cómo estas personas se han visto afectadas por la COVID-19 en lo que respecta a sus condiciones de trabajo, ingresos y bienestar, y cómo las plataformas y los poderes públicos han respondido para protegerlas de los efectos negativos de la pandemia. Además, es crucial rastrear los impactos a nivel macroeconómico en la propia economía de plataformas. ¿Ha permitido la COVID-19 que las empresas propietarias de plataformas se infiltren aún más en el funcionamiento de nuestra infraestructura social y económica esencial? ¿Ha impulsado la pandemia la demanda y la oferta de trabajadoras y trabajadores de plataformas? ¿Ha erosionado aún más los logros clave del movimiento laboral? A la inversa, la cuestión de si la economía de plataformas puede ofrecer soluciones a las múltiples crisis a las que nos enfrentamos se hizo más acuciante cuando el desempleo masivo se cernió sobre las sociedades como resultado del impacto en las empresas de los confinamientos prolongados. ¿Puede dicha economía ayudar a resolver los problemas de desempleo, en ventajosa sinergia con la revolución del trabajo flexible que ha desencadenado la pandemia?

El rápido ascenso y difusión de las plataformas tipo Uber se ha basado sobre todo en su capacidad para eludir la responsabilidad y la rendición de cuentas por las condiciones y los riesgos experimentados por sus trabajadoras y trabajadores (Woodcock y Graham 2020). Sin embargo, a principios de 2021 (año anterior a la redacción del presente artículo), las plataformas se vieron sometidas a una mayor presión pública y política para que modificaran sus políticas y protegieran a las y los trabajadores de los riesgos inmediatos que planteaba la pandemia, así como de la vulnerabilidad estructural que dichas plataformas habían identificado, explotado y perpetuado hasta entonces. Al tomar medidas para proteger a las y los trabajadores —por ejemplo, proporcionando equipos de protección personal (EPP), subsidios por enfermedad e indemnizaciones por pérdida de ingresos—, dichas plataformas corren el riesgo de socavar el lábil ámbito jurídico y retórico en el que han fundado sus modelos de negocio.

Las investigadoras e investigadores del proyecto Fairwork[1] realizamos un seguimiento de las condiciones de trabajo en las plataformas digitales de trabajo localizado[2] con respecto a determinados criterios de trabajo justo, que se han ido desarrollando en colaboración con las y los trabajadores y otras partes interesadas desde 2018. Cuando se declaró la pandemia de COVID-19 pudimos adaptar esta metodología y aprovechar nuestras redes de investigación ya establecidas para abordar algunas de las cuestiones que acabamos de plantear. A partir de abril de 2020 creamos y mantuvimos una base de datos de las iniciativas adoptadas por las plataformas frente a la pandemia, centrándonos en la medida en que preveían o establecían condiciones de trabajo justas y seguras en el contexto de la pandemia. Las y los investigadores del proyecto recopilamos estos datos mediante el análisis de documentos y el contacto directo con las plataformas y con las y los trabajadores de las mismas. Revisamos las políticas que habían publicado, así como noticias e informes analíticos, las respuestas de los sindicatos y otros grupos de personas trabajadoras, y la información proporcionada por las plataformas en sus sitios web y a través de sus canales de difusión pública. También nos pusimos en contacto con todas las plataformas incluidas en nuestra base de datos y las invitamos a que nos proporcionaran información sobre sus políticas y operaciones. A partir de una revisión de las políticas de más de 191 plataformas en 43 países, consideramos si las respuestas de las plataformas ante la COVID-19 han creado mejores condiciones de trabajo y han reducido la precariedad de su personal. Sobre la base

[1] Véase www.fair.work.

[2] Es decir, las plataformas a través de las que se proponen servicios vinculados a una determinada localización geográfica, como la entrega de comida, los servicios de limpieza y el transporte por carretera, que tienen lugar en una ubicación física concreta. Por el contrario, en el presente artículo no consideramos las plataformas de trabajo en línea, es decir, aquel que puede realizarse desde cualquier lugar (por ejemplo, el hogar), como la transcripción, el etiquetado de imágenes o la asistencia virtual.

de los criterios de trabajo justo en las plataformas establecidos en el proyecto, creamos una tipología de respuestas de políticas bajo las categorías de salario justo, condiciones justas y gestión justa, con el fin de ayudarnos a comprender en qué medida la pandemia ha cuestionado o modificado las convenciones que sustentan la economía de plataformas.[3]

Nuestras conclusiones son alentadoras, ya que apuntan a que se han tomado algunas medidas positivas para proteger a las y los trabajadores de plataformas durante este periodo. Sin embargo, también identificamos algunas deficiencias significativas en materia de protección y la posibilidad de que se perennice el trabajo precario en las plataformas como consecuencia de la pandemia, habiéndose puesto de manifiesto ejemplos de oportunismo ante la crisis. Por último, examinamos diversos acontecimientos políticos derivados de la pandemia, entre ellos, ejemplos de resistencia laboral consolidada, pero también de plataformas que presionan con éxito para lograr un cambio legislativo a su favor.

La inesperada conmoción que ha supuesto la pandemia representa un punto de inflexión en materia de condiciones laborales en la economía de plataformas, y la situación podría evolucionar hacia dos posibles escenarios extremos. En uno de ellos, las empresas de plataformas podrían aprovechar el momento de incertidumbre para afianzar aún más un modelo de explotación y expandirlo a nuevos sectores. Ciertamente, ya estamos viendo pruebas de ello en muchos lugares. En un segundo escenario, la COVID-19 podría servir de catalizador para un futuro más justo del trabajo en plataformas, lo cual requeriría, en nuestra opinión, que las entidades legisladoras respondan a los llamamientos de las y los trabajadores de plataformas y aprovechen el momento para colmar definitivamente las lagunas que las plataformas han utilizado para privar a las y los trabajadores de unas condiciones laborales dignas. Dado que los gobiernos se están moviendo con mayor licencia pública tanto para proteger los medios de vida actuales como para permitir la creación de otros nuevos, este es un momento crucial para reglamentar las plataformas digitales de trabajo para un futuro laboral más justo.

El resto del presente artículo se organiza como sigue. En la segunda sección se exploran los efectos del primer año de la pandemia en los mercados laborales y las consecuencias para las y los trabajadores de plataformas digitales. En la tercera se analizan las dificultades a las que experimentan estas personas para acceder a las ayudas gubernamentales debido a su situación laboral. En la cuarta se evalúan las respuestas de las plataformas para abordar los riesgos a los que se enfrentan sus trabajadoras y trabajadores en relación con los criterios de trabajo justo de Fairwork. En la quinta sección se presenta evidencia de un aumento de la acción colectiva de las y los trabajadores de plataformas, contrastada, en la sexta sección, con los esfuerzos de las plataformas por negar

[3] Debido en parte a la escasez de datos disponibles públicamente sobre el número y la distribución de las y los trabajadores de plataformas, nuestro estudio no establece distinciones entre las plataformas ni las clasifica según el número de trabajadoras y trabajadores inscritos en ellas. Cada plataforma, independientemente de su tamaño, se cuenta como una unidad, aunque al identificar la muestra nos centramos en las plataformas más destacadas de cada país incluido en el estudio.

sus responsabilidades. En la última sección resumimos nuestras conclusiones y proponemos vías de acción oportunas para proteger los derechos de las y los trabajadores de las plataformas.

### 2. COVID-19 y la digitalización del trabajo

En el presente artículo se analizan las tendencias observadas durante el primer año de la pandemia de COVID-19. En aquel momento se preveía que las repercusiones sociales y económicas fueran graves, pero el panorama aún estaba en ciernes. En enero de 2021, más de 90 millones de personas se habían contagiado de COVID-19 y más de 2 millones habían muerto, cifras que desde entonces han aumentado exponencialmente. En su informe sobre perspectivas de la economía mundial de abril de 2020, el Fondo Monetario Internacional (FMI) preveía que el crecimiento mundial en dicho año cayera hasta el –3 por ciento, y que el crecimiento en las economías avanzadas se situara en el –6,1 por ciento (FMI 2020). La pérdida acumulada del producto interior bruto (PIB) mundial prevista durante 2020 y 2021 era de unos 9 billones de dólares de los Estados Unidos (Gopinath 2020), lo que representaba la peor recesión económica desde la Gran Depresión y la primera vez desde los años treinta del siglo pasado que los países de ingreso elevado, mediano y bajo estaban simultáneamente en recesión. Basándose en un análisis de la disminución de la producción (entre el 50 y el 100 por ciento) de los sectores más inmediatamente afectados por los paros generalizados, la Organización para la Cooperación y el Desarrollo Económicos (OCDE) predijo un efecto inicial en el PIB de las economías del Grupo de los Siete[4] de entre el 20 y el 25 por ciento (OCDE 2020).

Aunque la economía mundial comenzó a recuperarse en 2021, el proceso se ha visto frenado por el aumento de la inflación y el inicio de la guerra en Ucrania en 2022. Los efectos de estas crisis en los mercados financieros, la actividad económica y la vida social siguen acumulándose a medida que la pandemia se prolonga hasta su tercer año. El presente artículo ofrece una instantánea de las primeras repercusiones de la pandemia en los trabajadores de plataformas, centrándose en el impacto de las primeras medidas de contención adoptadas, en particular los confinamientos y la designación de ciertos tipos de trabajo como «esenciales». Sin embargo, reconocemos que algunos de los riesgos y presiones presentes al principio de la pandemia han disminuido a medida que los confinamientos se han vuelto menos habituales. Por el contrario, otros riesgos se han agudizado debido a acontecimientos más recientes, como el aumento de la inflación, que ha afectado de manera desproporcionada a las y los trabajadores con bajos ingresos o con empleos precarios y a los países de ingreso bajo y mediano.

En cuanto al mercado laboral, a finales de mayo de 2020 la OIT (2020) informó de que el 94 por ciento de las y los trabajadores del mundo vivían en países que imponían algún tipo de cierre de los lugares de trabajo en un intento de controlar la propagación del virus. Alrededor del 20 por ciento de las y los trabajadores del mundo vivían en países que exigían el cierre de todos los centros de trabajo salvo si se trataba de trabajadoras y trabajadores «esenciales»

[4] Alemania, Canadá, Estados Unidos, Francia, Italia, Japón y Reino Unido.

(aunque la definición de «esencial» varía de un país a otro), y casi el 70 por ciento vivían en países que exigían cierres de lugares de trabajo para determinados sectores o categorías de trabajadores. La OIT también estimó una disminución de las horas de trabajo de más del 10 por ciento en el segundo trimestre de 2020 en relación con el último trimestre de 2019, lo que equivale a 305 millones de empleos a tiempo completo en todo el mundo (suponiendo una semana laboral de 48 horas). Según estas cifras, se estima que las Américas y Europa y Asia Central han sufrido las mayores pérdidas de horas trabajadas a nivel mundial (13,1 y 12,9 por ciento, respectivamente, según OIT 2020).

Además, la crisis no afectó a todas las personas trabajadoras por igual ni de la misma manera. Mientras que para algunos grupos el trabajo desde el hogar se convirtió en una realidad que les permitía ahorrar en gastos de transporte o comida, otros se enfrentaron a graves consecuencias en forma de despidos, permisos no remunerados o acuerdos laborales cada vez más precarios. El cierre casi total amenazó a algunos sectores, como la hostelería, el turismo, el comercio minorista y el ocio, generando una pérdida generalizada de medios de vida. Las trabajadoras y trabajadores jóvenes experimentaron algunos de los peores efectos. En mayo de 2020, la OIT (2020) indicó que más del 15 por ciento de las y los jóvenes habían dejado de trabajar desde el inicio de la crisis de COVID-19, mientras que quienes seguían trabajando experimentaron, en promedio, una reducción de las horas de trabajo del 23 por ciento. A escala global, en abril de 2020, el 16,1 por ciento de las mujeres jóvenes y el 18,1 por ciento de los hombres jóvenes (de 18 a 29 años) habían dejado de trabajar. Ellos (46 por ciento) informaron de una reducción de ingresos con mayor frecuencia que ellas (38 por ciento, según OIT 2020).

Paralelamente, el distanciamiento social y los confinamientos periódicos exigieron a la sociedad adaptarse a una nueva normalidad en la que el contacto humano directo se minimizó o se eliminó por completo, lo que condujo a una aceleración de la digitalización de la economía (Baig et al. 2020). Esto incluyó un rápido aumento del uso de plataformas digitales, comercio electrónico, herramientas de vigilancia de las y los trabajadores, «trabajo en la nube» y trabajo por encargo (Sneader y Singhal 2020), lo que alteró las prácticas convencionales de gestión y organización, tradicionalmente basadas en la proximidad física y la observación (Malev 2020).

Las y los trabajadores de plataformas han mantenido servicios públicos esenciales durante la pandemia, como la entrega de alimentos y artículos de primera necesidad a quienes se autoaislaban o practicaban el distanciamiento social o la prestación de servicios de atención a quienes los necesitaban. Los pedidos de comidas o de alimentos a través de plataformas, los pagos sin dinero en efectivo, la entrega de paquetes sin contacto y el uso de plataformas o aplicaciones para contratar chóferes privados en lugar de utilizar el transporte público se han normalizado, incluso en países donde antes no era así. Según un informe prepandémico de la OCDE, las y los trabajadores de las plataformas representan entre el 0,3 (en los países en desarrollo) y el 0,5 (en el resto) de las y los trabajadores de todo el mundo, llegando hasta el 3 por ciento de la población en edad de trabajar en algunos países (Schwellnus et al. 2019). Sigue siendo muy difícil estimar las cifras globales de trabajadoras y trabajadores de plataformas —que dichas empresas no revelan—, pero es probable que estos porcentajes hayan aumentado aún más como resultado de la pandemia. Los confinamientos recurrentes y la recesión financiera han provocado, sin duda, que muchas personas en todo el mundo pierdan un empleo seguro y busquen como alternativa un trabajo a través de plataformas.

### 3. Cayendo en las grietas: acceso de las y los trabajadores de plataformas a la protección social

No está claro si el aumento de la oferta de trabajadoras y trabajadores de plataformas como consecuencia de la pandemia ha ido acompañado de un aumento de la demanda de su mano de obra, y esto habrá variado ciertamente según el sector. De hecho, observamos una marcada polarización en la economía de plataformas entre las y los trabajadores esenciales, que pueden haber visto aumentar su carga de trabajo —como los mensajeros de última milla y de entrega de alimentos—, y otras áreas, como el cuidado personal (estética) y los servicios domésticos, que en 2020 vieron una caída dramática y a veces legislada del volumen de negocio. Dentro del primer grupo, es posible que se hayan mantenido los ingresos, pero habrá sido a costa de una exposición continuada a los riesgos para la salud. En el Reino Unido, por ejemplo, el 28 por ciento de las y los trabajadores de plataformas encuestados declaró tener más trabajo del habitual en agosto de 2020. De ellos, el 78 por ciento consideraba que su salud corría un mayor riesgo mientras trabajaba, pero siguió trabajando de todos modos debido a la gran preocupación por perder sus ingresos (Blundell, Machin y Ventura 2020).

En los sectores de la economía de plataformas que experimentaron un descenso sustancial de la demanda observamos un descenso igualmente drástico de los ingresos: una encuesta mundial realizada durante los confinamientos más estrictos de marzo de 2020 reveló que el 70 por ciento de las y los trabajadores de plataformas no tenían ingresos y el 90 por ciento buscaba nuevas fuentes de ingresos (Moulds 2020). Una de las fuentes de apoyo económico a las que recurrieron estos trabajadores de plataformas fueron las ayudas gubernamentales. Esto era de esperar, dado que los gobiernos adoptaron una respuesta sin precedentes ante la COVID-19: en mayo de 2020, 190 países habían anunciado un total de casi 1000 programas de protección social para sus ciudadanas y ciudadanos, entre los que se incluyen muchas ayudas para complementar o sustituir los ingresos de las personas trabajadoras (Gentilini et al. 2020).

Sin embargo, al menos en las primeras etapas de la pandemia, las y los trabajadores de plataformas se encontraron con un problema de larga data al intentar acceder a estos programas. Sobre la base de los «indicios específicos» para determinar la existencia de una relación de trabajo estipulados en la Recomendación sobre la relación de trabajo, 2006 (núm. 198), de la OIT, o en la legislación derivada de la misma, las y los trabajadores de plataformas en muchos contextos pueden considerarse empleados de las plataformas (véase Bogg 2019; Heeks et al. 2020). Por ejemplo, muchas de estas personas son totalmente dependientes de las plataformas y tienen poco control sobre la forma en que realizan su trabajo o sobre el precio que pueden exigir por él, ambos indicios de la condición de empleado. Sin embargo, el estatus contractual de muchas de estas personas no es el de «empleado» de una plataforma, sino el de «contratista independiente».[5] Este estatus contractual ambiguo ha provocado que las y los trabajadores de muchos países no hayan podido beneficiarse de los programas gubernamentales aplicados para ayudar a los trabajadores durante la crisis (véase Mawii, Rathi y Tandon 2020). En el Reino Unido, el Self-Employment Income Support Scheme (plan gubernamental de apoyo a los ingresos de los trabajadores autónomos) no incluyó a quienes habían sido contratados después de abril de 2019 y no pudieron presentar los registros fiscales necesarios, o que obtuvieron menos de la mitad de sus ingresos del trabajo por cuenta propia (Belger 2020). Como señalan Lord et al. (2020), muchas trabajadoras y trabajadores de plataformas no podían acogerse a este régimen porque era muy poco probable que hubieran trabajado durante más de dos años. En Francia, las y los trabajadores de plataformas no se consideran oficialmente empleados y, por lo tanto, solo habrían podido optar a un pago único de 1500 euros, destinado específicamente a los «microemprendedores» (Apouey et al. 2020). Sudáfrica ofrece otro ejemplo de la importancia de la clasificación de las y los trabajadores de plataformas a la hora de acceder a las ayudas en el contexto de la pandemia. Si hubieran sido realmente contratistas independientes, habrían sido registrados formalmente como empresa, y podrían haber solicitado préstamos blandos en el marco del COVID-19 Debt Relief Fund (fondo gubernamental de alivio de la deuda covid-19) o la suscripción de préstamos ya existentes. Por otra parte, si las y los trabajadores de plataformas hubieran sido reconocidos como empleados y sus plataformas hubieran estado cotizando al fondo de seguro de desempleo del país, entonces dicha plataforma podría haberse acogido al COVID-19 Temporary Employee/Employer Relief Scheme (plan de ayuda temporal para empleados y empleadores), que proporcionaba ingresos de sustitución para las y los trabajadores despedidos como consecuencia de la pandemia. Como no se cumplía ninguna de las dos condiciones, las y los trabajadores de plataformas no tuvieron acceso a la ayuda gubernamental. Como consecuencia de estas dificultades, las encuestas realizadas en varios países mostraron que la falta de ingresos era la principal preocupación de las y los trabajadores de plataformas durante el primer semestre de 2020 (Flourish 2020a y 2020b; Fairwork 2020a).

A medida que la pandemia hacía estragos a lo largo de 2020, algunos gobiernos ampliaron las disposiciones de protección social para cubrir a las y los trabajadores de plataformas y anunciaron moratorias en el pago de préstamos y deudas.[6] Sin embargo, a pesar de la introducción de nuevas disposiciones, seguían existiendo importantes obstáculos para acceder a las ayudas públicas. Las y los trabajadores de plataformas afirmaron que las moratorias sirvieron para

[5] La cuestión de si las y los trabajadores de plataformas tienen una relación de trabajo y, por lo tanto, si han sido mal clasificados contractualmente por las plataformas, es una fuente de controversia jurídica sustancial y de debate legislativo en todo el mundo. La respuesta a esta pregunta es sin duda compleja y depende de las especificidades de los sectores, las jurisdicciones, los acuerdos de subcontratación y otros factores contextuales. No pretendemos zanjar esta cuestión aquí, sino más bien llamar la atención sobre la lógica débil y contradictoria que las plataformas están promoviendo en la actualidad, y las consiguientes oportunidades de activismo a favor de las y los trabajadores y de intervención de las instancias legisladoras.

[6] Véase FMI, «Policy Responses to COVID-19», Policy Tracker, https://www.imf.org/en/Topics/imf-and-covid19/Policy-Responses-to-COVID-19/.

reducir gastos como los reembolsos de los préstamos para automóviles o motocicletas utilizados para el trabajo de transporte o reparto (Flourish 2020b). Sin embargo, se tuvo noticia de acreedores que redujeron los pagos en lugar de suspenderlos por completo, como pretendía la moratoria, o bien que simplemente ignoraron las nuevas normas (Prawira Adjie 2020; Mawii 2020; Ustek-Spilda, Heeks y Graham 2020). Las y los trabajadores también informaron de las dificultades para acceder a los regímenes de asistencia social debido a la lentitud de ejecución de los planes por parte de las administraciones públicas, a que carecían de la documentación necesaria o a que los regímenes solo cubrían a las y los ciudadanos nacionales o inmigrantes registrados formalmente (Fairwork 2020a; Pacis 2020). En los Estados Unidos, por ejemplo, los trabajadores y trabajadoras cubiertos por la US Coronavirus Aid, Relief, and Economic Security Act[7] experimentaron importantes retrasos en la recepción de la ayuda (Romo 2020). En la India, ciertos grupos pudieron recibir ayuda alimentaria o financiera del Gobierno, pero muchos otros se encontraron con barreras de acceso (Flourish 2020b). Dado que el trabajo de plataforma tiende a desarrollarse (ex profeso) en los vacíos normativos, incluso cuando las instancias públicas han hecho todo lo posible para incluir a estos trabajadores y trabajadoras en los regímenes de protección social, a veces ha resultado extremadamente difícil establecer los parámetros de quién tiene derecho a la asistencia y aplicar las disposiciones adecuadas para llegar a ellos. El resultado ha sido una asistencia poco sistemática, que se ha quedado muy lejos de las protecciones integrales de que disponen las trabajadoras y trabajadores de otros sectores, clasificados como empleados.

En ocasiones, estos problemas han creado incluso dificultades para que las y los trabajadores pudieran cubrir sus necesidades básicas, como se refleja en las entrevistas que realizamos a personas que trabajan en plataformas de Sudáfrica:

> No tenemos comida. No puedo alimentar a mis hijos. Todavía estamos esperando los paquetes de comida del Gobierno, no estamos seguros de que los vayamos a recibir (trabajador de plataforma en Ciudad del Cabo, abril de 2020).
>
> El Gobierno debe ayudarnos también con la comida, pero yo soy un extranjero aquí en Sudáfrica, así que no creo que el Gobierno pueda ayudarnos con la comida (trabajador de una plataforma en Ciudad del Cabo, abril de 2020).

Así, en sus inicios, la pandemia creó dos categorías de trabajadores de plataformas: los no esenciales, que no podían seguir trabajando ni tenían acceso a las ayudas económicas, y los esenciales, que no tenían más remedio que seguir arriesgando su salud yendo a trabajar porque tampoco tenían acceso a dicha protección. Las plataformas han creado activamente las condiciones para que esto ocurra al construir sus modelos de negocio sobre un tipo de trabajadoras y trabajadores precarios, es decir, nominalmente independientes, pero con una agencia real muy limitada. La experiencia de estas trabajadoras y trabajadores de plataformas durante la pandemia ha demostrado hasta qué punto las plataformas ejercen un control similar al de un empleador sobre las condiciones, la seguridad y el bienestar de quienes trabajan para ellas. Dejando a un lado el estatus jurídico —que es ambiguo, polémico y dependiente de la jurisdicción—,

[7] Congreso de los Estados Unidos, Coronavirus Aid, Relief, and Economic Security Act, Public Law 116–136, 27 March 2020, 134 STAT. 281.

la creación y extracción de valor de las plataformas se basa en el trabajo y el riesgo asumido por sus trabajadoras y trabajadores, con quienes establece una relación de dependencia. Por lo tanto, sostenemos que las plataformas tienen la responsabilidad cívica fundamental de respetar los derechos básicos de las y los trabajadores a la seguridad y salud en el trabajo, y de protegerlos de los riesgos asociados a su trabajo, riesgos que la COVID-19 ha incrementado significativamente. Esta responsabilidad se hace aún más acuciante cuando no pueden acceder a los derechos y salvaguardias que la normativa estatal otorga a las personas consideradas empleadas.

### 4. Respuestas de las plataformas ante los riesgos creados por la pandemia para las y los trabajadores

Ante el aumento de la inseguridad y la precariedad del trabajo en plataformas, el presente artículo explora lo que estas han hecho para mitigar los riesgos derivados de la pandemia de COVID-19 y para mejorar las condiciones de trabajo. Hemos creado una tipología que pretende dar cuenta de todos los tipos de políticas aplicadas como respuesta a la crisis de COVID-19 y, además, nos permite examinar hasta qué punto el virus ha modificado las convenciones que sustentan la economía de plataformas. Estructuramos nuestro análisis en torno a los principios de salario justo, condiciones justas y gestión justa del proyecto Fairwork (Graham et al. 2020) (véase el anexo 1 para más detalles).[8]

Los resultados de nuestra investigación muestran que algunas plataformas han modificado considerablemente sus políticas para mitigar los riesgos asociados a la pandemia, pero son minoría (para una visión general de las políticas promulgadas, véase el anexo 2). En algunos casos, especialmente en la categoría de condiciones justas —relacionadas con la salud y la seguridad—, estos cambios parecen derivarse de la normativa gubernamental de cumplimiento obligatorio, más que de la voluntad de las propias plataformas. En general, encontramos importantes carencias en las respuestas de las plataformas a la pandemia, y creemos que hay que hacer mucho más para garantizar unas condiciones de trabajo justas en la economía de plataformas, tanto en el contexto de la pandemia como posteriormente.

#### 4.1. Remuneración justa

La pérdida de salario ha sido uno de los principales riesgos para las y los trabajadores de plataformas durante la pandemia, y no es de extrañar que fuera la cuestión que más preocupaba a la mayoría de las personas entrevistadas para el presente estudio. Sin embargo, los esfuerzos de las plataformas para

[8] Además de estas tres categorías, Fairwork ha formulado otros dos principios importantes de trabajo justo en la economía de plataformas que nos sirven de referencia para evaluarlas: contrato justo y representación justa (Graham et al. 2020). Sin embargo, se han excluido como categorías en el presente análisis porque son menos pertinentes en relación con las medidas inmediatas de mitigación de riesgos, por lo que no se incluyeron en la tipología de políticas de plataformas para las que recabamos evidencia.

proporcionar apoyo financiero no fueron en general muy abundantes. De las 191 plataformas analizadas, solo 21 aportaron pruebas de que habían proporcionado a las y los trabajadores algún tipo de compensación directa por la pérdida de ingresos debida a las menores oportunidades de trabajo. Más de la mitad de esas plataformas son grandes empresas multinacionales (Uber, Uber Eats, Glovo, Amazon y Grab) que tienen capacidad financiera para compensar directamente a las y los trabajadores por la pérdida de ingresos. Sin embargo, varias plataformas locales más pequeñas también ofrecieron formas de apoyo a los ingresos. Sin duda, los recursos financieros de que disponen las plataformas multinacionales son de magnitud mucho mayor, por lo que tienen mayor capacidad para establecer mecanismos de protección de los ingresos. Entre las más pequeñas, cabe citar la plataforma sudafricana de limpieza doméstica SweepSouth, que estableció un fondo al que la clientela y otros donantes podían contribuir para apoyar a las y los trabajadores durante el confinamiento.[9]

En un pequeño número de plataformas encontramos evidencia de otras formas de apoyo financiero. Dado que muchos trabajadores y trabajadoras de plataformas contraen préstamos para comprar equipos de trabajo o alquilar sus vehículos, ya sea directamente a las plataformas o a través de empresas con las que las plataformas han establecido acuerdos de financiación, varias plataformas garantizaron el aplazamiento o la reducción de los reembolsos. Sin embargo, solo 34 plataformas, en su mayoría del sector del transporte y el reparto, ofrecieron esta forma de apoyo financiero (entre ellas, Ola en la India, Lyft en los Estados Unidos, Gojeck en Indonesia, Glovo en Argentina, España, Francia e Italia, y Uber en varios mercados).

Aún más limitada fue la oferta de préstamos para las y los trabajadores que se enfrentan a dificultades financieras específicamente como resultado de la pandemia, aunque cabe citar las iniciativas de Uber en Jordania, Grab en Singapur y Didi Chuxing en China. Por último, solo 21 plataformas parecen haber aplicado políticas para mantener un nivel de actividad empresarial que pudiera ayudar a mantener los ingresos de las y los trabajadores, como por ejemplo la renuncia o el aplazamiento de las cuotas de la clientela para animarla a inscribirse en una plataforma y la creación de nuevos servicios, como en los casos de las plataformas de transporte individual de pasajeros que ofrecieron servicios de reparto y las plataformas de reparto de comidas preparadas que empezaron a ofrecer también reparto de alimentos.

### 4.2. Condiciones justas

Los riesgos de seguridad y salud asociados a la COVID-19 generaron un amplio abanico de políticas de las plataformas en respuesta, que van desde la protección de las y los trabajadores y la clientela contra el contagio hasta el apoyo a las y los trabajadores enfermos. Hemos clasificado este tipo de respuestas en dos subcategorías: *medidas preventivas* y *medidas relacionadas con la enfermedad*. En comparación con las disposiciones de las otras dos categorías (remuneración justa y gestión justa), las disposiciones sobre seguridad y salud han experimentado la evolución más drástica en el transcurso de la pandemia y se han observado en

[9] Véase https://campaign.sweepsouth.com/ss-covid19-fund-2020/.

el mayor número de plataformas. Al principio de la pandemia, la mayoría de las plataformas se abstenían de proporcionar a sus trabajadoras y trabajadores cualquier tipo de protección en materia de seguridad y salud (para evitar que se pusiera en duda su clasificación como contratistas independientes), pero en los meses siguientes, necesitaban demostrar a la clientela y a las autoridades que aplicaban procedimientos de seguridad estrictos para mantener su licencia social para operar.

#### 4.2.1. Medidas preventivas

El cambio a servicios con distancia social fue una de las respuestas más comunes entre las plataformas, pues más del 60 por ciento de ellas introdujeron alguna forma de servicio sin contacto. Estas políticas incluían la obligatoriedad de los pagos sin efectivo para reducir el manejo de monedas y billetes. En el sector del transporte individual de pasajeros, donde el distanciamiento social es más difícil, varias plataformas previeron la instalación gradual de paneles de protección entre conductores y clientes. Con el tiempo, una gran parte de las plataformas proporcionó a las y los trabajadores equipos de protección personal (EPP) gratuitos, el 63 por ciento distribuyó o reembolsó la compra de desinfectantes, y el 48 por ciento proporcionó o reembolsó la compra de mascarillas. En agosto de 2020, más de 50 países habían hecho obligatorio el uso de mascarillas en espacios públicos (*Al Jazeera* 2020), lo que obligó a muchas plataformas a garantizar su cumplimiento durante la prestación de servicios. Además, el 27 por ciento de las plataformas aplicaron medidas de saneamiento para proteger a las y los trabajadores y a la clientela, incluyendo la desinfección de vehículos y almacenes, o el cierre de los centros. Otra medida relativamente común fue la difusión de directrices para evitar el contagio: casi el 50 por ciento de las plataformas proporcionaron a sus trabajadoras y trabajadores algún tipo de orientación al respecto, aunque en la mayoría de los casos se limitaron a los consejos sanitarios generales emitidos por los gobiernos.

A pesar de la adopción de toda esta serie de políticas, los datos demuestran que la aplicación de las medidas de protección no siempre fue eficaz; por ejemplo, varias plataformas proporcionaron EPP solo de forma puntual y no permanente, y otras —entre las que se encuentran algunas grandes plataformas de transporte y reparto, como Uber, Uber Eats, Deliveroo, Rappi y Ola— no proporcionaron EPP a todos los trabajadores y trabajadoras o experimentaron dificultades y retrasos a la hora de reembolsarles las compras de EPP (Anderson 2020; Hadi 2020). Además, no todas las políticas preventivas estaban destinadas a proteger a las y los trabajadores. Si bien la mayoría de las plataformas de reparto introdujeron servicios sin contacto, estos generalmente incluían solo el contacto con la o el cliente final, pero no el contacto en restaurantes o almacenes, por ejemplo. Otras políticas tenían como objetivo proteger únicamente a la clientela, como hacer obligatorios los escaneos de temperatura para las y los trabajadores, o suspender sus cuentas en caso de contraer la COVID-19.

#### 4.2.2. Medidas relacionadas con la enfermedad

Algo más del 50 por ciento de las plataformas ofrecieron algún tipo de ayuda económica a las y los trabajadores que contraían la COVID-19. Solo la pequeña minoría de plataformas que clasifica a sus trabajadoras y trabajadores como empleados o contratistas dependientes pudo recurrir a planes de subsidio de enfermedad patrocinados por los gobiernos, mientras que las demás implementaron fondos de apoyo financiero privados. Aunque el monto de las ayudas varía sustancialmente según las plataformas y los países, por lo general fueron relativamente bajas en comparación con los ingresos medios de las y los trabajadores de plataformas y se proporcionaron como «tarifa plana». Solo ocho plataformas garantizaron los niveles de ingresos anteriores.

Aunque se crearon varios regímenes de ayuda económica por enfermedad, el acceso a los mismos resultó a menudo problemático. En varios casos, como los de las y los trabajadores de Deliveroo en el Reino Unido y los de Amazon en los Estados Unidos, dicho acceso fue imposible (Manthorpe 2020; Slisco 2020). En otros casos, la prueba requerida para acceder al plan, como un certificado médico, era imposible de obtener para quienes debían autoaislarse.

### 4.3. Gestión justa

Solo una minoría de plataformas parece haber aplicado cambios positivos en las políticas de gestión durante la pandemia. En la arquitectura de muchas plataformas, el número de tareas asignadas a un trabajador o trabajadora o los incentivos de bonificación dependen en gran medida de la tasa de aceptación previa de la persona, de su calificación o de otras métricas basadas en las tareas ya realizadas. Por lo tanto, el hecho de no poder mantener el mismo nivel de actividad durante la pandemia podría dificultar sus perspectivas laborales y salariales futuras. Cincuenta y nueve plataformas, entre ellas Uber y Careem en varios países, Hermes en el Reino Unido y Amazon en los Estados Unidos, introdujeron políticas para garantizar que las y los trabajadores no pierdan las bonificaciones o los incentivos en caso de no poder trabajar durante un determinado periodo.

Otro riesgo al que se enfrentaron las y los trabajadores de plataformas pertenecientes a grupos minoritarios desde el inicio de la pandemia fue el aumento de la discriminación, ya que en muchos casos se trató de explicar la propagación del virus culpando a determinados grupos sociales en diversos contextos. Al revisar las medidas adoptadas por las plataformas a partir de marzo de 2020 para proteger a las y los trabajadores del acoso y la discriminación descubrimos que 28 plataformas —entre ellas Uber, Uber Eats y Lyft— hicieron declaraciones públicas en las que afirmaban que no se toleraría la discriminación contra ningún trabajador o trabajadora (por ejemplo, por motivos de raza o de nacionalidad).

Los cambios observados en las políticas de gestión no fueron necesariamente positivos para las y los trabajadores, y varias de las medidas más comunes suponían problemas de privacidad. Como ya se ha mencionado, varias plataformas introdujeron escáneres de temperatura para las y los trabajadores; otras los obligaron a hacerse «selfies» para demostrar que llevaban mascarillas o guantes, o utilizaron aplicaciones de rastreo de contactos (Moghe y Rathee 2020; Clarance 2020). A menudo, los datos recabados se compartían directamente con la clientela, sin tener en cuenta las normas de protección de datos. Aunque estas políticas pretendían garantizar la seguridad y salud en general y tranquilizar a la clientela, también aumentaban de facto el ya elevado nivel de vigilancia de las y los trabajadores por parte de la plataforma, con poca o ninguna comunicación o control de cómo se gestionaban y utilizaban sus datos.

Las medidas relativamente inmediatas, o provisionales, expuestas anteriormente fueron esenciales para proteger a las y los trabajadores durante las fases agudas de la crisis. Sin embargo, de nuestro análisis se desprende que en 2020 la mayoría de las y los trabajadores de plataformas del mundo no se beneficiaron ni de la asistencia gubernamental ni del apoyo de las plataformas para amortiguar el golpe económico de los confinamientos, o para hacer frente a riesgos específicos como el contagio. La cuestión que nos planteamos ahora es cómo la provisión o ausencia de apoyo a las y los trabajadores de plataformas durante la pandemia puede haber modificado las normas y convenciones de las relaciones laborales en la economía de plataformas. En la siguiente sección exploramos las respuestas de las y los trabajadores y la creciente incidencia de sus iniciativas de resistencia; después examinamos algunas implicaciones de la pandemia para el modelo de negocio de las plataformas digitales de trabajo.

### 5. Voz y resistencia de las y los trabajadores de plataformas durante la pandemia

En 2020, a medida que la pandemia dejaba cada vez más patente la profunda asimetría de poder que caracteriza las relaciones laborales de la economía de plataformas, se produjo un aumento de la acción colectiva por parte de las y los trabajadores de la misma. De hecho, el aumento de los niveles de inseguridad física y económica, unido a la ausencia de medidas de protección adecuadas por parte de las plataformas, provocó huelgas, protestas y acciones judiciales por parte de las y los trabajadores en todo el mundo. En Sudáfrica, por ejemplo, las y los conductores de la aplicación de transporte individual Bolt organizaron varios paros en septiembre y octubre de 2020 en protesta por la disminución de los salarios y las deficientes medidas de seguridad (Mlamla 2020; Malinga 2020). Sin embargo, a pesar de que se produjeron varias acciones similares en todo el mundo, las plataformas, en su mayoría, ignoraron las demandas de las y los trabajadores. Algunas incluso intentaron suprimir las expresiones de la voz colectiva de las y los trabajadores. Ese fue el caso en la India, donde la plataforma de reparto Zomato bloqueó los perfiles de las y los repartidores que participaron en las huelgas (Fairwork 2020b). Aunque las condiciones y reivindicaciones expresadas en estas acciones dirigidas por las y los trabajadores eran específicas de cada contexto, colectivamente hablan de la influencia de las condiciones pandémicas en el creciente poder de organización y visibilidad de las y los trabajadores de plataformas.

Las huelgas de repartidores en América Latina en el segundo semestre de 2020 ofrecen un ejemplo especialmente ilustrativo de las estrategias innovadoras que las y los trabajadores emplearon para movilizarse contra las prácticas injustas de la economía de plataformas. El 1 de julio de 2020, las y los repartidores de varias plataformas importantes en las principales ciudades de la Argentina, el Brasil, Chile, el Ecuador y México organizaron una serie de huelgas masivas coordinadas que atrajeron la atención internacional. La disminución de los salarios y la falta de EPP, especialmente en los primeros meses de la pandemia, fueron algunas de las principales preocupaciones de los miles de trabajadoras y trabajadores que participaron en las numerosas protestas en toda la región. Estas protestas fueron facilitadas, al menos en parte, por los mismos procesos de digitalización frente a los que resistían. Las redes sociales fueron una herramienta clave utilizada por las y los trabajadores para movilizarse y obtener el apoyo de la opinión pública mediante la concienciación de las y los consumidores acerca de su precaria situación (Pskowski y Vilela 2020). Los sucesos de América Latina constituyen incluso un hito histórico, en la medida en que representan «el primer ejemplo real de un movimiento de huelga internacional y sectorial en la economía de plataformas» (Howson et al. 2020, 20).

Un ejemplo de éxito de organización de las y los trabajadores para mejorar su protección se dio en el Reino Unido. En noviembre de 2020, el Tribunal Superior de Inglaterra y Gales falló a favor del Independent Workers' Union of Great Britain (IWGB), un sindicato de trabajadoras y trabajadores de plataformas que había solicitado un examen judicial urgente sobre el cumplimiento por parte de las empresas de plataformas de su obligación legal de proporcionar EPP (Croft 2020). El IWGB representa a unas 5000 personas que trabajan como conductoras y mensajeras, y sostenía que el Gobierno no había aplicado correctamente las directivas de la UE que darían a las y los trabajadores de la economía de plataformas los mismos derechos de seguridad y salud que a las personas consideradas empleadas. También mantuvo que la cuestión se había vuelto aún más apremiante en el contexto de la pandemia.

En medio de la creciente inseguridad y el caos creados por la pandemia de COVID-19, las y los trabajadores también se movilizaron en torno a las continuas batallas legales sobre su condición de empleados. Ese fue el caso de la campaña «No on Prop. 22» (No a la Propuesta 22) en California. Se trataba de una iniciativa de ley presentada en noviembre de 2020 por una coalición de poderosas plataformas —Uber, Lyft, Postmates, Instacart y DoorDash— en respuesta al proyecto de ley 5 (AB5).[10] La AB5 es una ley laboral aprobada por la Asamblea del Estado de California en 2019 que exige que todos los trabajadores y trabajadoras de plataformas de dicho estado sean reclasificados como empleados y, por lo tanto, que las empresas de plataformas les proporcionen el conjunto completo de prestaciones propias de los empleados, incluyendo el pago de horas extraordinarias, la licencia por enfermedad pagada y la atención médica proporcionada por el empleador. La Propuesta 22 pretendía eximir a las empresas de transporte y reparto basadas en aplicaciones del cumplimiento de la AB5 y, en su lugar, establecer un «ámbito intermedio», de forma que las y los trabajadores accedieran a algunas protecciones y prestaciones, pero sustancialmente menores que las que les corresponden en virtud de la AB5 (Steward et al. 2020).

La campaña liderada por las y los trabajadores contra la Propuesta 22 recibió niveles de apoyo sin precedentes por parte de los movimientos sindicales tanto en los Estados Unidos como a escala internacional. Las plataformas habían conseguido inicialmente que se aprobara su propuesta durante las elecciones presidenciales estadounidenses de 2020, tras una campaña publicitaria muy bien financiada (Hussain, Bhuiyan y Menezes 2020), pero la medida fue posteriormente anulada, al ser declarada anticonstitucional por el Tribunal Superior de Alameda de California (Tusk 2021). Aunque la Propuesta 22 fue finalmente anu-

[10] State of California, Assembly Bill No. 5, aprobado el 18 de septiembre de 2019.

lada, los acontecimientos que rodearon su adopción inicial son un recordatorio aleccionador del inmenso poder de presión de las plataformas multinacionales de trabajo. También generaron un llamamiento a la acción para que las asociaciones de trabajadores y los grupos políticos impidan que las plataformas reproduzcan el modelo en otros lugares, y sugieran alternativas más justas, como la reciente Propuesta de Directiva de la UE para la mejora de las condiciones laborales en plataformas digitales (Chaibi 2020).[11] En febrero de 2021, el Tribunal Supremo del Reino Unido confirmó la reivindicación de la condición de empleado (y los derechos y protecciones que conlleva, incluido el salario mínimo) de un grupo de conductores y conductoras de Uber representados por el sindicato App Drivers and Couriers (Butler 2021). Aunque se trata de una victoria importante para el poder de las y los trabajadores de plataformas en el Reino Unido y a escala internacional, Uber sigue sin cumplir plenamente la sentencia, según la cual han de concederse a las y los conductores los derechos que otorga el estatus de empleado durante todas las horas que estén conectados y disponibles para trabajar. La plataforma solo aceptó garantizar el salario mínimo y otras prestaciones durante el tiempo en que las y los trabajadores estuvieran activamente realizando un trabajo.

Está claro que la pandemia ha aumentado la presión sobre las y los trabajadores de plataformas y ha exacerbado su precariedad y su inseguridad hasta un extremo insostenible en muchos casos. Esto, a su vez, ha impulsado una resistencia colectiva a una escala nunca vista en la economía de plataformas (Howson et al. 2020). También ha dado lugar a nuevas estrategias de las plataformas para preservar sus modelos de negocio explotadores. Todavía está por ver si los movimientos de las y los trabajadores pueden consolidarse lo suficiente como para obtener beneficios duraderos.

### 6. ¿Con las y los trabajadores o con las y los accionistas? La pandemia expone la lógica contradictoria del modelo de negocio de las plataformas

Desde su creación, las plataformas laborales se han posicionado deliberadamente —tanto retórica como jurídicamente— como «empresas tecnológicas» o «mercados digitales» que se dedican a poner en contacto a quienes quieren vender diversos servicios con quienes quieren comprarlos (Woodcock y Graham 2020). De este modo han creado una distancia artificial entre ellas y quienes ofrecen trabajo a través de sus interfaces digitales. En la mayoría de las jurisdicciones, a pesar del control sustancial que ejercen sobre los ingresos y las condiciones de trabajo de sus trabajadoras y trabajadores, las plataformas han mantenido firmemente que no son empleadoras y que las y los trabajadores de plataformas no son, por tanto, sus empleados. Al clasificarlos contractualmente

[11] Comisión Europea, Propuesta de Directiva del Parlamento Europeo y del Consejo relativa a la mejora de las condiciones laborales en el trabajo en plataformas digitales, Bruselas, 9 de diciembre de 2021, COM(2021) 762 final.

como contratistas independientes, trabajadores por cuenta propia o similares, las plataformas han podido eludir en gran medida las costosas obligaciones de la entidad empleadora, como las cotizaciones a la seguridad social. Además, las y los trabajadores de plataformas —a diferencia de la mayoría de los trabajadores formales— no tienen derecho a las protecciones laborales que tanto ha costado conquistar, como el salario mínimo o la licencia pagada por enfermedad.

Sin embargo, este modelo de negocio ha sido objeto de un escrutinio cada vez más crítico en los últimos años, a medida que los estudios y los medios de comunicación han ido centrando la atención del público en los riesgos del trabajo en plataformas (Schor 2020). El modus operandi de las plataformas también se ha vuelto más difícil de justificar y mantener a la luz de las crecientes —y en muchos casos, totalmente evitables— tragedias humanas resultantes de la pandemia de COVID-19. Aunque el trabajo en plataformas siempre se ha caracterizado por sus malas condiciones laborales (véase, por ejemplo, Cant 2019), la crisis sanitaria ha añadido una nueva y acuciante dimensión a este problema. Además, la cuestión de salvaguardar la salud de las y los trabajadores durante esta pandemia —dado que su trabajo implica contactos personales en buena parte de los casos, como los servicios de reparto y de transporte individual de pasajeros— está directamente relacionada con la de salvaguardar la salud de toda la población.

A pesar de las medidas positivas que las plataformas han adoptado en respuesta a la crisis pandémica, también hemos visto resistencia a la aplicación de ciertas medidas básicas y observamos que muchos trabajadores y trabajadoras han quedado sin protección. En parte, esto debe atribuirse al hecho de que tales medidas iban en contra de la lógica que ha alimentado el tremendo éxito de la economía de plataformas desde sus inicios. Aunque la mayoría de las protecciones otorgadas a estos trabajadores han sido básicamente fragmentarias, inadecuadas y de difícil acceso, al promulgarlas, las plataformas están admitiendo *implícitamente* su responsabilidad por los riesgos financieros y sanitarios que esos trabajadores corren. Como hemos argumentado recientemente con respecto a Uber, «para una empresa que ha insistido repetidamente en que no "emplea" a sus conductores, estas disposiciones representan un giro. Reconocen implícitamente que Uber tiene un control significativo sobre las condiciones, la salud e incluso la supervivencia de dichas personas, lo que la convierte en mucho más que la propietaria de un mercado digital» (Katta et al. 2020, 205). En esencia, la pandemia ha puesto a las plataformas laborales digitales contra una incómoda pared: simultáneamente afirman no ser empleadoras, mientras que en la práctica introducen medidas parecidas a la protección del empleo. El contradictorio limbo que ocupan actualmente las empresas de la economía de plataformas está dando pábulo a los litigios a favor de las y los trabajadores sobre cuestiones de clasificación laboral, así como una oportunidad para que las instancias legisladoras se aseguren de que las plataformas asumen su responsabilidad proporcionando a las y los trabajadores protecciones duraderas e institucionalizadas incluso más allá de la pandemia.

Aunque hay motivos para el optimismo sobre las oportunidades que ofrecen a los litigantes y a las entidades legisladoras las políticas de COVID-19 de las plataformas, también hay motivos de preocupación debido a una tendencia opuesta: aunque las plataformas han hecho algunas concesiones a sus trabajadores en forma de subsidios por enfermedad y compensación por pérdida de ingresos, también han tenido un éxito considerable a la hora de influir en las instancias legisladoras para que desarrollen una reglamentación que afiance y permita su estrategia empresarial a expensas de los derechos y las protecciones de quienes trabajan para ellas. Las disputas políticas y jurídicas durante la pandemia ponen de manifiesto el creciente dominio de las plataformas en la formación de la opinión pública, y cómo esto se traduce en la reglamentación. En los Estados Unidos, el poder de presión de las plataformas se puso de manifiesto en el éxito de la campaña «Yes on Prop. 22» (Sí a la Propuesta 22), que a su vez dio a las plataformas un impulso financiero con capital de riesgo y clientela (Ustek-Spilda et al. 2020).

Uber, Lyft y sus aliados gastaron una asombrosa cantidad de dinero para defender la Propuesta 22. Los 200 millones de dólares invertidos en la campaña fueron la mayor suma gastada en una iniciativa de ley en la historia de los Estados Unidos. En cambio, quienes defendían a las y los trabajadores recaudaron aproximadamente una décima parte de esa cantidad. Uber, en particular, hizo un uso liberal de la interfaz de su plataforma para compartir mensajes políticos y propaganda de la campaña con conductores y usuarios. Cuando se aprobó la medida, las acciones de Uber y Lyft subieron un 18 por ciento y un 22 por ciento, respectivamente, lo que representa una ganancia mucho mayor que los 200 millones de dólares invertidos. Aunque la Propuesta 22 fue posteriormente declarada inconstitucional, este momento histórico no indica un consenso inminente sobre el papel y la responsabilidad de las plataformas; más bien amplía los ámbitos de conflicto preexistentes entre las instancias legisladoras, las empresas de plataformas y las y los trabajadores. La gran influencia que pudieron ejercer las plataformas también puede derivarse del hecho de que los servicios que facilitan, como los de reparto, han demostrado ser esenciales para la respuesta de la sanidad pública a la pandemia.

### 7. Conclusión: ¿Qué nueva normalidad creará la pandemia en la economía de las plataformas?

Nuestra investigación pone de manifiesto que algunas plataformas han intervenido cuando sus trabajadoras y trabajadores se enfrentaban a riesgos relacionados con la pandemia que no estaban cubiertos por las protecciones sociales o gubernamentales. Fundamentalmente, esto demuestra que tienen un poder definitorio sobre la seguridad y las condiciones laborales de esas trabajadoras y trabajadores (Ustek-Spilda, Heeks y Graham 2020). Sin embargo, nuestros datos también demuestran que la mayoría de las plataformas no han estado a la altura de la responsabilidad que corresponde a ese poder, por lo que, en ausencia de protecciones laborales e intervenciones gubernamentales, muchos trabajadores y trabajadoras de plataformas han caído en el olvido. En el presente artículo sugerimos algunas razones estructurales subyacentes a la reticencia de las plataformas a instituir medidas de protección y garantizar los derechos laborales durante la pandemia. Nuestra investigación más amplia en el marco del proyecto Fairwork también ha demostrado que el poder de las plataformas se ejerce de diferentes maneras en distintos contextos. En algunos casos han adoptado estrategias alternativas al afianzamiento de la clasificación de los trabajadores como independientes, como se ha visto en California. En Alemania, por ejemplo, las leyes laborales y de transporte público municipales han obligado a Uber a adaptar su estrategia encargando a intermediarios privados que empleen a sus conductores (Fairwork 2020c). Sin embargo, estos acuerdos de empleo subcontratado también pueden dejar a las y los trabajadores en una situación precaria y vulnerable. Varios conductores en Alemania nos dijeron que, a pesar de estar empleados por un intermediario, ganaban menos del salario mínimo. Los conductores y conductoras tampoco sabían si estaban cubiertos por un seguro de accidentes (Fairwork 2020c). Estos hallazgos han sido corroborados con nuestra investigación en Sudáfrica (Fairwork 2020d) y en la India (Fairwork 2020b). En todos estos contextos hemos observado que las plataformas interactúan con los entornos sociales, políticos y económicos de maneras distintas, con el objetivo común de evadir o eludir los marcos normativos que les son desfavorables y socavar la resistencia colectiva de las y los trabajadores.

Sin embargo, hay varios casos que indican posibles formas de domar el poder de las plataformas (Woodcock y Graham 2020; Ustek-Spilda et al. 2020). Junto a la importancia del poder asociativo de las y los trabajadores, cuya eficacia ha quedado demostrada durante la pandemia con el ejemplo del éxito de las acciones judiciales de los sindicatos en el Reino Unido, vemos otras perspectivas complementarias de cambio positivo. Por ejemplo, Evgeny Morozov (2020) sostiene que la tecnología de las plataformas digitales se ha convertido en algo tan importante para mediar en los procesos económicos y dirigir sus resultados distributivos, que estas infraestructuras digitales deberían tratarse como un bien público. Una vía para la propiedad compartida y la gobernanza de las infraestructuras de plataformas se encuentra en el naciente movimiento de plataformas cooperativas (Scholz 2016). Sin embargo, aunque son prometedoras, estas cooperativas penan por el momento para lograr cierta posición competitiva frente a las empresas de plataformas ya establecidas, cuyo enfoque ha seguido siendo ganar cuotas de mercado casi monopólicas, incluso cuando esto significa subvencionar servicios con una pérdida financiera continua. Dado este desequilibrio de mercado, otra forma de controlar el poder asimétrico de las plataformas podría ser a través de mecanismos antimonopolio. Como sostiene Sanjukta Paul, una estrategia de este tipo podría contribuir a reasignar gobernanza «a los actores más pequeños y alejarla de los dominantes», de modo que «un municipio podría gestionar la aplicación y coordinar públicamente el mercado, teniendo en cuenta el interés público» (entrevista en Angwin 2020).

Sin embargo, estas intervenciones se quedan en pura teoría sin la implicación de los gobiernos. Incluso en este momento en que la pandemia está exacerbando las desigualdades «mediadas» por las plataformas y su poder corporativo, estamos siendo testigos de cómo las plataformas empiezan a cumplir una función cada vez más infraestructural. Sin embargo, al no haber rendición pública de cuentas, esto conduce a una falta de gobernanza democrática en la economía de plataformas. Como hemos mostrado, durante la pandemia los mercados financieros han recompensado a las plataformas por eludir con éxito las responsabilidades jurídicas y sociales. Por lo tanto, ahora es más importante que nunca revisar nuestra idea de lo que son las plataformas y de lo que han de hacer en un mundo pospandémico. En el presente artículo hemos señalado un posible punto de partida, desde el que las protecciones introducidas durante la pandemia pueden utilizarse tanto para establecer una nueva base de referencia para las protecciones de los trabajadores de plataformas como para cuestionar los argumentos de las plataformas respecto a su nivel de responsabilidad jurídica hacia sus trabajadoras y trabajadores. Si no se sigue prestando atención a la forma en que los mecanismos actuales de la economía de plataformas se estructuran intrínsecamente en contra de las y los trabajadores, y si no se plantea un reto social y político a la altura, puede ser imposible evitar un futuro del trabajo que simplemente acelere la competencia a la baja en lo que respecta a las condiciones de trabajo y a la protección de las trabajadoras y trabajadores.

### Bibliografía citada

*Al Jazeera*. 2020. «Which Countries Have Made Wearing Face Masks Compulsory?». 17 de agosto de 2020. https://www.aljazeera.com/news/2020/04/countries-wearing-face-masks-compulsory-200423094510867.html/.

Anderson, Tom. 2020. «A Delivery Rider Accuses Deliveroo of Not Taking Workers' Health Seriously». *Canary*, 21 de abril de 2020. https://www.thecanary.co/exclusive/2020/04/21/a-delivery-rider-accuses-deliveroo-of-not-taking-workers-health-seriously/.

Angwin, Julia. 2020. «The Antitrust Case for Gig Worker Rights». *Markup*, 7 de noviembre de 2020. https://www.getrevue.co/profile/themarkup/issues/the-antitrust-case-for-gig-worker-rights-289982.

Apouey, Bénédicte, Alexandra Roulet, Isabelle Solal y Mark Stabile. 2020. «Gig Workers during the COVID-19 Crisis in France: Financial Precarity and Mental Well-Being». *Journal of Urban Health* 97 (6): 776-795.

Baig, Aamer, Bryce Hall, Paul Jenkins, Eric Lamarre y Brian McCarthy. 2020. «The COVID-19 Recovery Will Be Digital: A Plan for the First 90 Days». *McKinsey Digital*, 14 de mayo de 2020. https://www.mckinsey.com/business-functions/mckinsey-digital/our-insights/the-covid-19-recovery-will-be-digital-a-plan-for-the-first-90-days#.

Belger, Tom. 2020. «Coronavirus: Gig Workers Sue UK Government over Support for Self-Employed». *Yahoo Finance UK*, 8 de mayo de 2020. https://uk.finance.yahoo.com/news/coronavirus-uber-legal-action-gig-workers-sue-uk-government-rights-230055092.html.

Berg, Janine, Marianne Furrer, Ellie Harmon, Uma Rani y M. Six Silberman. 2019. *Las plataformas digitales y el futuro del trabajo: cómo fomentar el trabajo decente en el mundo digital*. Ginebra: OIT.

Blundell, Jack, Stephen Machin y Maria Ventura. 2020. «Covid-19 and the Self-Employed: Six Months into the Crisis», Centre for Economic Performance Working Paper No. 012. Londres: London School of Economics and Political Science.

Bogg, Alan. 2019. «Taken for a Ride: Workers in the Gig Economy». *Law Quarterly Review* 135 (abril): 219-226.

Butler, Sarah. 2021. «Uber Drivers Entitled to Workers' Rights, UK Supreme Court Rules». *Guardian*, 19 de febrero de 2021. https://www.theguardian.com/technology/2021/feb/19/uber-drivers-workers-uk-supreme-court-rules-rights.

Cant, Callum. 2019. *Riding for Deliveroo: Resistance in The New Economy*. Cambridge: Polity Press.

Chaibi, Leïla. 2020. «A Proposal to Legislate for the Rights of Platform Workers». *Social Europe*, 16 de noviembre de 2020. https://www.socialeurope.eu/a-proposal-to-legislate-for-the-rights-of-platform-workers/.

Clarance, Andrew. 2020. «Aarogya Setu: Why India's Covid-19 Contact Tracing App Is Controversial». *BBC News*, 15 de mayo de 2020. https://www.bbc.co.uk/news/world-asia-india-52659520/.

Croft, Jane. 2020. «UK Gig Economy Workers Win High Court Battle over PPE». *Financial Times*, 13 de noviembre de 2020. https://www.ft.com/content/b7b2dc47-305f-4aee-a74e-0ad16c71d63c.

Fairwork. 2020a. «The Gig Economy and Covid-19: Looking Ahead». Septiembre de 2020. Oxford.

— 2020b. *Fairwork India Ratings 2020: Labour Standards in the Platform Economy*. Oxford.

— 2020c. *Fairwork Germany Ratings 2020: Labour Standards in the Platform Economy*. Oxford.

— 2020d. «Gig Workers, Platforms and Government during Covid-19 in South Africa». Mayo de 2020. Oxford.

Flourish. 2020a. «The Digital Hustle: Gig Worker Financial Lives Under Pressure». Indonesia Spotlight September 2020. Washington.

— 2020b. «The Digital Hustle: Gig Worker Financial Lives under Pressure». India Spotlight September 2020. Washington.

FMI (Fondo Monetario Internacional). 2020. «Perspectivas de la economía mundial: El Gran Confinamiento». Abril de 2020. Washington.

Forde, Chris, Mark Stuart, Simon Joyce, Liz Oliver, Danat Valizade, Gabriella Alberti, Kate Hardy et al. 2017. «The Social Protection of Workers in the Platform Economy». Estudio para la Comisión de Empleo y Asuntos Sociales del Parlamento Europeo. Bruselas: Parlamento Europeo. [Resumen en español en https://www.europarl.europa.eu/RegData/etudes/STUD/2017/614184/IPOL_STU(2017)614184(SUM01)_ES.pdf.]

Gentilini, Ugo, Mohamed Almenfi, Pamela Dale, John Blomquist, Harish Natarajan, Guillermo Galicia, Robert Palacios y Vyjayanti Desai. 2020. «Social Protection and Jobs Responses to COVID-19: A Real-Time Review of Country Measures», Living Paper Version 10, 22 de mayo de 2020. Washington: Banco Mundial.

Gopinath, Gita. 2020. «El Gran Confinamiento: La peor desaceleración económica desde la Gran Depresión». *Diálogo a fondo* (blog del FMI), 14 de abril de 2020. https://blog-dialogoafondo.imf.org/?p=13190.

Graham, Mark, y Mohammad Amir Anwar. 2019. «The Global Gig Economy: Towards a Planetary Labour Market?». *First Monday* 24 (4). https://doi.org/10.5210/fm.v24i4.9913.

Graham, Mark, Jamie Woodcock, Richard Heeks, Paul Mungai, Jean-Paul Van Belle, Darcy du Toit, Sandra Fredman, Abigail Osiki, Anri van der Spuy y Six M. Silberman. 2020. «The Fairwork Foundation: Strategies for Improving Platform Work in a Global Context». *Geoforum* 112 (junio): 100-103.

Hadi, Abdurzak. 2020. «I'm an Uber Driver Who Watched a Young Colleague Die from Coronavirus. Now I'm Suing the Government for Failing to Protect Us». *Independent*, 16 de abril de 2020. https://www.independent.co.uk/voices/coronavirus-deaths-uber-ayub-akthar-cab-drivers-key-workers-a9468146.html.

Heeks, Richard, Mark Graham, Paul Mungai, Jean-Paul Van Belle y Jamie Woodcock. 2020. «Systematic Evaluation of Platform Work against Decent Work Standards; Development of a New Framework and Application in the Global South», Digital Development Working Paper Series, No. 85. Manchester: Centre for Digital Development.

Howson, Kelle, Funda Ustek-Spilda, Rafael Grohmann, Nancy Salem, Rodrigo Carelli, Daniel Abs, Julice Salvagni et al. 2020. «"Just Because You Don't See Your Boss, Doesn't Mean You Don't Have a Boss": Covid-19 and Gig Worker Strikes across Latin America». *International Union Rights* 27 (3): 20-28.

Hussain, Suhauna, Johana Bhuiyan y Ryan Menezes. 2020. «How Uber and Lyft Persuaded California to Vote Their Way». *Los Angeles Times*, 13 de noviembre de

2020. https://www.latimes.com/business/technology/story/2020-11-13/how-uber-lyft-doordash-won-proposition-22/.

Katta, Srujana, Adam Badger, Mark Graham, Kelle Howson, Funda Ustek-Spilda y Alessio Bertolini. 2020. «(Dis)embeddedness and (De)commodification: COVID-19, Uber, and the Unravelling Logics of the Gig Economy». *Dialogues in Human Geography* 10 (2): 203-207.

Lord, Carolynne, Oliver Bates, Adrian Friday, Fraser McLeod, Andy Oakey, Tom Cherrett y Toni Martinez-Sykora. 2020. «Written Evidence Submitted by the FlipGig project (COV0123)». Convocatoria de presentación de pruebas a la Comisión de Empresas, Energía y Estrategia Industrial (Business, Energy and Industrial Strategy Committee), junio de 2020. Londres: Parlamento del Reino Unido.

Malev, Milko. 2020. «Is the Coronavirus Pandemic an Engine for the Digital Transformation?». *DMEXCO*, 3 de abril de 2020. https://dmexco.com/stories/is-the-coronavirus-pandemic-an-engine-for-the-digital-transformation/.

Malinga, Sibahle. 2020. «Bolt Drivers, Operators March against "Exploitation"». *ITWeb*, 23 de septiembre de 2020. https://www.itweb.co.za/content/o1Jr5qx9Z39vKdWL/.

Manthorpe, Rowland. 2020. «Coronavirus: Deliveroo Riders Say They Can't Access Promised Hardship Fund». *Sky News*, 18 de marzo de 2020. https://news.sky.com/story/coronavirus-deliveroo-riders-say-they-cant-access-promised-hardship-fund-11959632/.

Mawii, Zothan. 2020. «Covid-19 and Relief Measures for Gig Workers in India». *Centre for Internet & Society*, 14 de abril de 2020. https://cis-india.org/raw/zothan-mawii-covid-19-and-relief-measures-for-gig-workers-in-india.

Mawii, Zothan, Aayush Rathi y Ambika Tandon. 2020. «From Health and Harassment to Income Security and Loans, India's Gig Workers Need Support». *Centre for Internet & Society*, 30 de abril de 2020. https://cis-india.org/raw/gig-workers-need-support.

Mlamla, Sisonke. 2020. «Uber, Bolt Drivers in SA Protest Over Low Wages and Exploitation». *IOL*, 13 de octubre de 2020. https://www.iol.co.za/capeargus/motoring/uber-bolt-drivers-in-sa-protest-over-low-wages-and-exploitation-7e45c944-bb6f-428a-8842-fbeac2b6a56a#:~:text=Boltpor ciento20Southpor ciento20Africapor ciento20managerpor ciento20Gareth,abilitypor ciento20topor ciento20hailpor ciento20apor ciento20ride/.

Moghe, Ritwik, y Sunil Rathee. 2020. «Facing up to a Pandemic: A Look at Swiggy's Efforts to Counter the COVID-19 Threat». *Swiggy Bytes*, 8 de mayo de 2020. https://bytes.swiggy.com/facing-up-to-a-pandemic-a-look-at-swiggys-efforts-to-counter-the-covid-19-threat-7b9ec0bd7e5f.

Morozov, Evgeny. 2020. «Digital Public Infrastructure: The Social Democratic Project of the Twenty-First Century», Wiso Direkt, 08/2020. Bonn: Friedrich-Ebert-Stiftung.

Moulds, Josephine. 2020. «Gig Workers Among the Hardest Hit by Coronavirus Pandemic». *World Economic Forum*, 21 de abril de 2020. https://www.weforum.org/agenda/2020/04/gig-workers-hardest-hit-coronavirus-pandemic/.

OCDE (Organización para la Cooperación y el Desarrollo Económicos). 2020. «Evaluating the Initial Impact of COVID-19 Containment Measures on Economic Activity». 10 de junio de 2020. París.

OIT. 2020. «Observatorio de la OIT: La COVID-19 y el mundo del trabajo». Cuarta edición, 27 de mayo de 2020. Ginebra.

Pacis, Jessamine. 2020. «A Catch-22 for On-Demand Cleaners in the Philippines». *Bot Populi*, 1 de mayo de 2020. https://botpopuli.net/a-catch-22-for-on-demand-cleaners-in-the-philippines/.

Prawira Adjie, Moch. Fiqih Prawira. 2020. «App-Based "Ojek" Drivers Demand Compensation as New Social Restrictions Bar Them from Taking Passengers». Jakarta Post, 8 de abril de 2020. https://www.thejakartapost.com/news/2020/04/07/app-based-ojek-drivers-demand-compensation-as-new-social-restrictions-bar-them-from-taking-passengers.html/.

Pskowski, Martha, y Rafael Vilela. 2020. «"They Aren't Anything Without Us": Gig Workers Are Striking throughout Latin America». *Vice*, 11 de agosto de 2020. https://www.vice.com/en/article/jgxazk/they-arent-anything-without-us-gig-workers-are-striking-throughout-latin-america/.

Romo, Vanessa. 2020. «Self-Employed and Gig Workers Face Long Waits for Coronavirus Relief Checks». *NPR*, 17 de abril de 2020. https://www.npr.org/2020/04/17/836670935/self-employed-and-gig-workers-face-long-waits-for-coronavirus-relief-checks/.

Scholz, Trebor. 2016. *Platform Cooperativism: Challenging the Corporate Sharing Economy*. Nueva York: Rosa Luxemburg Stiftung. [Traducido al español por Victor Guillamon. 2016. *Cooperativismo de plataforma: desafiando la economía colaborativa corporativa*. Barcelona: Publicaciones Dimmons.]

Schor, Juliet B. 2020. *After the Gig: How the Sharing Economy Got Hijacked and How to Win It Back*. Oakland: University of California Press.

Schwellnus, Cyrille, Assaf Geva, Mathilde Pak y Rafael Veiel. 2019. «Gig Economy Platforms: Boon or Bane?». OECD Economics Department Working Papers No. 1550. París: OCDE.

Slisco, Aila. 2020. «Amazon Warehouse Workers Say Company Isn't Actually Providing Promised Paid Sick Leave». *Newsweek*, 8 de abril de 2020. https://www.newsweek.com/amazon-warehouse-workers-say-company-isnt-actually-providing-promised-paid-sick-leave-1496965/.

Sneader, Kevin, y Shubham Singhal. 2020. «The Future Is Not What It Used to Be: Thoughts on the Shape of the Next Normal». *McKinsey & Company*, 14 de abril de 2020. https://www.mckinsey.com/featured-insights/leadership/the-future-is-not-what-it-used-to-be-thoughts-on-the-shape-of-the-next-normal.

Stanford, Jim. 2017. «The Resurgence of Gig Work: Historical and Theoretical Perspectives». *Economic and Labour Relations Review* 28 (3): 382-401.

Steward, Shelly, Pablo Aguera Reneses, Srujana Katta y Mark Graham. 2020. «California's Proposition 22 Presents an Alarming Turning Point in Labor Law». *Fairwork*, 28 de octubre de 2020. https://fair.work/en/fw/blog/proposition-22/.

Tusk, Bradley. 2021. «A California Judge Just Struck Down Prop 22: Now What?». *TechCrunch*, 24 de agosto de 2021. https://techcrunch.com/2021/08/24/a-california-judge-just-struck-down-prop-22-now-what/?.

Ustek-Spilda, Funda, Fabian Ferrari, Matt Cole, Pablo Aguera Reneses y Mark Graham. 2020. «The Infrastructural Power of Platform Capitalism». Social Europe, 16 de diciembre de 2020. https://socialeurope.eu/the-infrastructural-power-of-platform-capitalism.

Ustek-Spilda, Funda, Richard Heeks y Mark Graham. 2020. «Covid-19: Who Will Protect Gig Workers, if Not Platforms?» *Social Europe*, 28 de mayo de 2020. https://www.socialeurope.eu/covid-19-who-will-protect-gig-workers-if-not-platforms/.

Woodcock, Jamie, y Mark Graham. 2020. *The Gig Economy: A Critical Introduction*. Cambridge: Polity.

## Anexo 1

### Lista de políticas de las plataformas en respuesta a la pandemia de COVID-19

| Tipo de política | Subcategoría | Acción de la plataforma |
|---|---|---|
| **1. Salario justo** | Indemnización por pérdida de salario | Pagos complementarios para compensar la pérdida de ingresos, incluido el aumento de los salarios |
| | Moratorias financieras | Préstamos de emergencia |
| | | Solicitar la reducción de los costes de alquiler de vehículos u otros a las empresas de alquiler |
| | | Suspensión del reembolso de préstamos |
| | | Aplazamiento de los gastos de préstamo/alquiler |
| | Intentos de compensar las pérdidas con nuevas estrategias de ingresos | Ampliación del alcance del servicio para aumentar el volumen de negocio tanto para la plataforma como para los trabajadores/as (por ejemplo, los servicios de transporte individual de pasajeros añaden servicios de reparto; los de reparto de comidas añaden reparto de alimentos) |
| | | Renuncia a las cuotas de la clientela para intentar mantener el volumen de negocio |
| | | Detener la incorporación de nuevos trabajadores/as para preservar los ingresos de los actuales |
| **2a. Condiciones justas (prevención)** | Protección física (trabajadores/as) | Entrega sin contacto, incluso sin firma y exigir pago sin efectivo |
| | | Barreras físicas instaladas en los vehículos |
| | | Suspender las cuentas de los clientes/as diagnosticados con COVID-19 |
| | | Saneamiento adicional a cargo de la plataforma, por ejemplo, de vehículos, almacenes, centros de distribución, etcétera. |
| | | Gestión sin contacto mediante el cierre de los centros físicos |
| | | Destruir toda la mercancía sin envasar para evitar contagios |
| | | Distanciamiento social/otras medidas en el tramo inicial de la cadena de suministro (preparación de alimentos y recogida/envasado de comestibles, etc.) |
| | Protección física (consumidores/as y población en general) | Suspender las cuentas de los conductores/as diagnosticados con COVID-19 |
| | | Prohibición de viajar de conformidad con las últimas directrices dadas por las autoridades locales/nacionales |
| | | Suspensión de algunos servicios |
| | Protección personal | Suministro de desinfectante (gel, espray) a los trabajadores/as |
| | | Suministro de mascarillas a los trabajadores/as |
| | | Instrucciones para seguir las mejores prácticas de higiene |
| | | Uso obligatorio de EPP, con control de las prácticas de higiene de los trabajadores/as |
| | Información de seguridad contra el virus | Suministro de información sobre prácticas sanitarias contra el virus |
| | | Formación sobre seguridad contra el virus |

### Lista de políticas de las plataformas en respuesta a la pandemia de COVID-19 *(fin)*

| Tipo de política | Subcategoría | Acción de la plataforma |
|---|---|---|
| **2a. Condiciones justas (prevención) *(fin)*** | Atención de salud | Revisiones médicas, inclusive medición de temperatura |
| | | Acceso a asesoramiento sanitario general |
| | | Acceso a asesoramiento sanitario personal (pagado/ proporcionado por la plataforma) |
| **2b. Condiciones justas (enfermedad)** | Pagos por enfermedad | Indemnización para los trabajadores/as enfermos o en cuarentena obligatoria (14 días) |
| | | Tarifa salarial habitual para los trabajadores/as enfermos o en cuarentena obligatoria (14 días) |
| | | Fondo de apoyo financiero (en principio, para la indemnización por enfermedad, pero ha de verificarse) |
| | | Se obliga a los trabajadores/as a firmar acuerdos de que la indemnización por enfermedad es específica del contexto pandémico y no cambia la situación (categoría) laboral |
| | Seguro | Provisión de seguro de salud por parte de la plataforma, o crédito en la cuenta de ahorro de salud |
| | Prestaciones por fallecimiento | Prestaciones por fallecimiento |
| **3. Contratos justos** | [No se han encontrado ejemplos de políticas] | [Se buscaban: cambios beneficiosos en las condiciones/ contratos/situación laboral de los trabajadores/as en respuesta a la COVID-19] |
| **4. Gestión justa** | Protección contra la penalización algorítmica | No se pierde el nivel de incentivos a pesar de un menor nivel de trabajo |
| | | Tiempo libre ilimitado y no remunerado sin penalización |
| | Medidas contra la discriminación | Declaración de la plataforma a los clientes de que no se tolerará la discriminación de determinados grupos de trabajadores/as durante la pandemia (por ejemplo, por motivos de higiene espurios) |
| **5. Representación justa** | [No se han encontrado ejemplos de políticas] | [Se buscaba: recepción formal de las reivindicaciones de los/as representantes de los trabajadores/as relacionadas con la COVID-19, diálogo con ellos y actuación al respecto] |
| | | Despido de trabajadores/as si participan en iniciativas de organización de trabajadores/as durante la pandemia |
| **Otros** | Donaciones | Donación sustancial u otro tipo de ayuda (por ejemplo, servicios gratuitos) a los servicios sanitarios nacionales, fondos comunitarios o similares |
| | Contratación | Iniciativa de contratación de trabajadores/as desempleados/as (no beneficia a los trabajadores ya en activo) |
| | Contracción de los servicios | Contracción del alcance de los servicios, por ejemplo, a la provisión de emergencias solamente |

Nota: En negro, las políticas en beneficio de los trabajadores/as; en gris, las que parecen ser para el beneficio de la plataforma pero con alguna ventaja potencial para los trabajadores/as; en azul claro, aquellas con probabilidades de tener consecuencias negativas para los trabajadores/as, y en azul oscuro, las que tienen consecuencias negativas directas para los trabajadores/as.

Fuente: Compilación propia basada en las categorías de trabajo justo de Fairwork (Graham et al. 2020).

## Anexo 2

### Plataformas que habían adoptado políticas clave para proteger a las y los trabajadores en septiembre de 2020

| | | Tipo de política (y subcategorías) | | | | | | | | | | |
|---|---|---|---|---|---|---|---|---|---|---|---|---|
| | | 1. Salario justo | | 2a. Condiciones justas (prevención) | | | | 2b. Condiciones justas (enfermedad) | | | 3. Gestión justa | |
| | Plataformas | Indemnización por pérdida de salario | Moratorias financieras | Protección física | Protección personal | Información sobre seguridad contra el virus | Atención de salud | Pagos por enfermedad | Seguro | Indemnizaciones por fallecimiento | Protección contra penalización algorítmica | Medidas contra la discriminación |
| Alemania | Uber | | ● | ● | ● | ● | ● | ● | | | ● | ● |
| | Clevershuttle | | | ● | ● | ● | ● | ● | ● | | ● | |
| | Lieferando | | | ● | ● | | | | | | ● | |
| Arabia Saudita | Careem | | ● | ● | ● | | | ● | | | | |
| | HungerStation | | | ● | | | | | | | | |
| | Lugmety | | | ● | ● | | | | | | | |
| | Carriage | | | ● | | | | | | | | |
| Argelia | Temtem | | | | ● | | | | | | | |
| | Yassir | | | ● | ● | | | | | | | |
| | Wesselni | | | | | | | | | | | |
| | Careem | | | ● | | | | ● | | | ● | |
| Argentina | Cabify | | | ● | ● | ● | ● | | | | | |
| | Beat | | | ● | ● | ● | ● | | | | ● | |
| | Glovo | ● | ● | ● | ● | ● | ● | ● | ● | | | |
| | Uber | | ● | ● | ● | ● | ● | ● | | | ● | ● |
| Armenia | Yandex.taxi | | | ● | ● | | | ● | | | | |
| | InDriver | | | | | | | | | | | |
| | GG | | | ● | ● | | | | | | | |
| | Menu.am | | | ● | ● | | | | | | | |
| | Sovats.am | | | ● | ● | | | | | | | |
| | Bolt | | | | ● | | | | | | | |
| Australia | Uber | | ● | ● | ● | ● | ● | ● | | | ● | ● |
| | Uber Eats | | | ● | ● | ● | ● | ● | | | ● | ● |
| | Deliveroo | | | ● | ● | ● | ● | ● | ● | ● | ● | |
| | Menulog | | | ● | | | | | | | | |
| | Coles | | | ● | ● | | | | | | | |
| Bahrein | Careem | | | ● | ● | | | ● | | | ● | |
| | Talabat | | | ● | | | ● | | | | | |
| | HungerStation | | | | | | | | | | | |

| | | Tipo de política (y subcategorías) | | | | | | | | | | |
|---|---|---|---|---|---|---|---|---|---|---|---|---|
| | | 1. Salario justo | | 2a. Condiciones justas (prevención) | | | | 2b. Condiciones justas (enfermedad) | | | 3. Gestión justa | |
| | Plataformas | Indemnización por pérdida de salario | Moratorias financieras | Protección física | Protección personal | Información sobre seguridad contra el virus | Atención de salud | Pagos por enfermedad | Seguro | Indemnizaciones por fallecimiento | Protección contra penalización algorítmica | Medidas contra la discriminación |
| Bangladesh | Uber | ● | ● | ● | ● | ● | ● | ● | | | ● | ● |
| | Sheba.xyz | | | ● | ● | ● | ● | | | | | |
| | Truck Lagbe | | | ● | ● | ● | ● | | | | | |
| Brasil | Uber | | ● | ● | ● | ● | ● | ● | | | ● | ● |
| | Cabify | | | ● | ● | ● | ● | | | | | |
| | iFood | | | ● | ● | ● | ● | ● | ● | | | |
| Chile | Uber | ● | ● | ● | ● | ● | ● | ● | | | ● | ● |
| | Uber Eats | | | ● | ● | ● | ● | ● | | | ● | ● |
| | Didi | | | ● | ● | | | ● | | | | |
| | Cabify | | | ● | ● | ● | ● | | | | | |
| | Beat | | | ● | ● | ● | ● | | | | ● | |
| | Rappi | | | ● | ● | | | ● | | | | |
| China | Didi Chuxing | | ● | ● | ● | ● | ● | ● | ● | ● | ● | |
| | Meituan Dache | | ● | ● | ● | ● | ● | | | | ● | |
| | Meituan Peisong | | | ● | ● | ● | ● | ● | | ● | ● | |
| | Ele (Fengniao Peisong) | | | ● | ● | | ● | ● | | ● | ● | |
| | FlashEx | | | ● | ● | | ● | ● | | ● | ● | |
| | Dianwoda | | | ● | ● | | ● | | | | ● | |
| | DADA Kuaisong | | | ● | ● | | ● | ● | | | ● | |
| Dinamarca | Wolt | | | ● | ● | ● | ● | ● | | | | |
| | Just Eat | | | ● | ● | ● | ● | ● | | | | |
| Egipto | Uber | ● | ● | ● | ● | ● | ● | ● | | | ● | ● |
| | Otlob | | | ● | ● | ● | | ● | | | | |
| | Swvl | | | ● | ● | ● | | ● | | | | |
| | Jumia | | | ● | | | | | | | | |
| Emiratos Árabes Unidos | Uber | | ● | ● | ● | ● | ● | ● | | | ● | ● |
| | Deliveroo | | | ● | ● | ● | ● | | | | | |
| | Talabat | | | ● | ● | | | ● | | | | |
| | Careem | | | ● | ● | | | ● | | | ● | |
| | Zomato | | | ● | ● | ● | | | | | | |

*(continúa en la página siguiente)*

| | | Tipo de política (y subcategorías) | | | | | | | | | | |
|---|---|---|---|---|---|---|---|---|---|---|---|---|
| | | 1. Salario justo | | 2a. Condiciones justas (prevención) | | | | 2b. Condiciones justas (enfermedad) | | | 3. Gestión justa | |
| | Plataformas | Indemnización por pérdida de salario | Moratorias financieras | Protección física | Protección personal | Información sobre seguridad contra el virus | Atención de salud | Pagos por enfermedad | Seguro | Indemnizaciones por fallecimiento | Protección contra penalización algorítmica | Medidas contra la discriminación |
| España | Glovo | | ● | ● | ● | ● | ● | ● | ● | | | |
| | Deliveroo | | | ● | ● | ● | ● | ● | | | | |
| | Just Eat | | | ● | ● | ● | ● | | | | | |
| | Uber | | ● | ● | ● | ● | ● | ● | | | ● | ● |
| | Uber Eats | | | ● | ● | ● | ● | ● | | | ● | ● |
| Estados Unidos | Amazon | ● | | ● | ● | | ● | ● | | | ● | |
| | Uber | | ● | ● | ● | ● | ● | ● | | | ● | ● |
| | Uber Eats | | | ● | ● | ● | ● | ● | | | ● | ● |
| | Lyft | | ● | ● | ● | ● | ● | ● | | | ● | ● |
| | Postmates | | | ● | | ● | ● | ● | ● | | | |
| | Doordash | | | ● | | ● | ● | ● | | | | |
| | Instacart | | | ● | ● | | | ● | | | | |
| | Grubhub | | | ● | ● | ● | ● | ● | | | ● | |
| Federación de Rusia | Yandex taxi | | | ● | ● | ● | ● | ● | | | | |
| | InDriver | | | ● | ● | | | | | | | |
| | Maxim | | | | ● | | | | | | | |
| | YouDo | | | | ● | | | | | | | |
| | Delivery Club | ● | | | | | | | | | | |
| | Bolt | | | | ● | | | | | | | |
| Filipinas | Grab | | ● | ● | ● | ● | ● | ● | | | | |
| Francia | Uber | | ● | ● | ● | ● | ● | ● | | | ● | ● |
| | Uber Eats | | | ● | ● | ● | ● | ● | | | ● | ● |
| | Deliveroo | | | ● | ● | ● | ● | ● | | | | |
| | Frichti | | | ● | ● | ● | ● | | | | | |
| | Glovo | | ● | ● | | | ● | ● | ● | | | |
| | Just Eat | | | ● | ● | ● | ● | | | | | |
| Georgia | Yandex taxi | | | ● | | | | ● | | | | |
| | GG | | | ● | | | | | | | | |
| | Glovo | | | ● | ● | ● | ● | ● | | | | |
| | Bolt | | | | ● | | | | | | | |

| | | Tipo de política (y subcategorías) | | | | | | | | | | |
|---|---|---|---|---|---|---|---|---|---|---|---|---|
| | | 1. Salario justo | | 2a. Condiciones justas (prevención) | | | | 2b. Condiciones justas (enfermedad) | | | 3. Gestión justa | |
| | Plataformas | Indemnización por pérdida de salario | Moratorias financieras | Protección física | Protección personal | Información sobre seguridad contra el virus | Atención de salud | Pagos por enfermedad | Seguro | Indemnizaciones por fallecimiento | Protección contra penalización algorítmica | Medidas contra la discriminación |
| Ghana | Uber | ● | ● | ● | ● | ● | ● | ● | | | ● | ● |
| | Bolt | | | ● | ● | | | | | | | |
| | Jumia Food | | | ● | ● | | ● | | | | | |
| India | Amazon | ● | | ● | ● | | ● | ● | | | ● | |
| | Uber | | ● | ● | ● | ● | ● | ● | | | ● | ● |
| | Ola | | ● | ● | ● | ● | ● | ● | | | | |
| | Zomato | | | ● | | ● | ● | ● | | | | |
| | Swiggy | | | ● | ● | ● | ● | ● | | | | |
| | BigBasket | | | ● | ● | | | | | | | |
| | Grofers | ● | | ● | ● | ● | ● | ● | | | | |
| | HouseJoy | | | | ● | ● | ● | | | | | |
| | Dunzo | | | ● | ● | ● | ● | | | | | |
| | UrbanClap | | | ● | ● | ● | ● | ● | ● | | | |
| | Flipkart | ● | | ● | ● | ● | ● | ● | ● | | | |
| | Shadowfax | | | ● | ● | ● | ● | ● | ● | | | |
| | Rapido | | | ● | ● | | ● | | ● | | | |
| Indonesia | Grab | | | ● | ● | | ● | ● | ● | | | |
| | Gojek | | ● | ● | ● | ● | ● | ● | | | | |
| Iraq | Careem | | | ● | ● | | | ● | | | ● | |
| Italia | Glovo | | ● | ● | ● | ● | ● | ● | ● | | | |
| | Deliveroo | | | ● | ● | ● | ● | ● | | | | |
| | Just Eat | | | ● | ● | ● | ● | | | | | |
| | Uber | | ● | ● | ● | ● | ● | ● | | | ● | ● |
| | Uber Eats | | | ● | ● | ● | ● | ● | | | ● | ● |
| Jordania | Careem | | | ● | ● | | | ● | | | ● | |
| | Uber | | ● | ● | ● | ● | ● | ● | | | ● | ● |
| | Talabat | | | ● | ● | | | | | | | |
| | Basket | | | ● | ● | | | | | | | |

*(continúa en la página siguiente)*

| | | Tipo de política (y subcategorías) | | | | | | | | | | |
|---|---|---|---|---|---|---|---|---|---|---|---|---|
| | | 1. Salario justo | | 2a. Condiciones justas (prevención) | | | | 2b. Condiciones justas (enfermedad) | | | 3. Gestión justa | |
| | Plataformas | Indemnización por pérdida de salario | Moratorias financieras | Protección física | Protección personal | Información sobre seguridad contra el virus | Atención de salud | Pagos por enfermedad | Seguro | Indemnizaciones por fallecimiento | Protección contra penalización algorítmica | Medidas contra la discriminación |
| Kenya | Jumia Food | | | ● | ● | | ● | | | | | |
| | Bolt | | | ● | ● | | | | | | | |
| | Lynk | | | | ● | | | | | | | |
| | Sendy | | | ● | ● | ● | ● | | ● | | | |
| | Uber | ● | ● | ● | ● | ● | ● | ● | | | ● | ● |
| Líbano | Careem | | | ● | | | | ● | | | ● | |
| | Zomato | | | | ● | | ● | | | | | |
| | Toters | | | ● | ● | | | | | | | |
| Marruecos | Careem | | | ● | ● | | | ● | | | ● | |
| | Yassir | | | | | | | | | | | |
| | Jumia | | | | ● | ● | ● | | | | | |
| | Jumia Food | | | | ● | ● | ● | | | | | |
| Nueva Zelandia | Uber | | | | ● | ● | ● | | | | | |
| | Uber Eats | | | ● | ● | ● | ● | | | | | |
| | DeliverEasy | | | ● | ● | | | | | | | |
| | MenuLog | | | ● | ● | | | | | | | |
| | Zoomy | | | ● | ● | ● | ● | | | | | |
| | Ola | | | | ● | ● | ● | | | | | |
| Nigeria | Jumia Food | | | ● | ● | | ● | | | | | |
| | Bolt | | | ● | ● | | | | | | | |
| | Uber | ● | ● | | ● | ● | ● | ● | | | ● | ● |
| Omán | Talabat | | | ● | | | | | | | | |
| Pakistán | Bykea | | | | ● | | ● | ● | | | | |
| | Foodpanda | | | ● | | | | | | | | |
| | Careem | | | ● | ● | ● | ● | ● | ● | | ● | |
| Qatar | Careem | | | ● | ● | | | ● | | | | |
| | Talabat | | | ● | ● | | | | | | | |
| | Carriage | | | | | | | | | | | |

| | | Tipo de política (y subcategorías) | | | | | | | | | | |
|---|---|---|---|---|---|---|---|---|---|---|---|---|
| | | 1. Salario justo | | 2a. Condiciones justas (prevención) | | | | 2b. Condiciones justas (enfermedad) | | | 3. Gestión justa | |
| | Plataformas | Indemnización por pérdida de salario | Moratorias financieras | Protección física | Protección personal | Información sobre seguridad contra el virus | Atención de salud | Pagos por enfermedad | Seguro | Indemnizaciones por fallecimiento | Protección contra penalización algorítmica | Medidas contra la discriminación |
| Reino Unido | Deliveroo | | | ● | ● | ● | ● | ● | | | | |
| | Uber | | ● | ● | ● | ● | ● | ● | | | ● | ● |
| | Uber Eats | | | ● | ● | ● | ● | ● | ● | | ● | ● |
| | JustEat | | | ● | ● | ● | ● | ● | | | | |
| | DPD | | ● | ● | ● | ● | ● | ● | | | | |
| | Hermes | | | ● | ● | ● | ● | ● | | | ● | |
| | Kapten | | | ● | ● | ● | ● | | | | | |
| | Syft | | | ● | ● | ● | ● | | | | | |
| | Bolt | | | ● | ● | ● | ● | ● | | | ● | |
| Serbia | Car.Go | | | ● | | | | | | | | |
| | alideda | | | ● | | | | | | | | |
| | Donesi | | | ● | | | | | | | | |
| | Bolt | | | | ● | | | | | | | |
| | Wolt | | | ● | ● | ● | ● | | | | | |
| Singapur | Grab | ● | ● | ● | ● | ● | ● | ● | | | ● | |
| | Deliveroo | | | ● | ● | ● | ● | ● | | | ● | |
| Sri Lanka | Uber | ● | ● | ● | ● | ● | ● | ● | | | ● | ● |
| | Pickme | | | | ● | ● | ● | | | | | |
| Sudáfrica | Uber | ● | ● | ● | ● | ● | ● | ● | | | ● | ● |
| | Uber Eats | ● | ● | ● | ● | ● | ● | ● | | | ● | ● |
| | Bolt | | | ● | ● | ● | ● | | | | | |
| | SweepSouth | ● | | ● | ● | ● | ● | ● | | ● | | |
| | OrderIn | | | | | | | | | | | |
| | Mr D | ● | | ● | ● | ● | ● | ● | | | | |
| | getTOD | | | ● | ● | | | | | | | |
| | NoSweat | | | | | | | | | | | |
| | M4Jam | ● | | | ● | ● | ● | | | | ● | |
| | Picup | | | ● | ● | | | | | | | |
| | Droppa | | | | | | | | | | | |
| | Secret Agent | | | | | | | | | | | |

*(continúa en la página siguiente)*

### Plataformas que habían adoptado políticas clave para proteger a las y los trabajadores en septiembre de 2020 *(fin)*

| | | Tipo de política (y subcategorías) | | | | | | | | | | |
|---|---|---|---|---|---|---|---|---|---|---|---|---|
| | | 1. Salario justo | | 2a. Condiciones justas (prevención) | | | | 2b. Condiciones justas (enfermedad) | | | 3. Gestión justa | |
| | Plataformas | Indemnización por pérdida de salario | Moratorias financieras | Protección física | Protección personal | Información sobre seguridad contra el virus | Atención de salud | Pagos por enfermedad | Seguro | Indemnizaciones por fallecimiento | Protección contra penalización algorítmica | Medidas contra la discriminación |
| Territorio palestino ocupado | Careem | | | ● | ● | | | ● | | | ● | |
| Túnez | Yassir | | | | | | | | | | | |
| | Founa | | | ● | ● | | | | | | | |
| | IntiGo | | | ● | ● | | | | | | | |
| | Jumia | | | ● | | | | | | | | |
| | Jumia Food | | | ● | | | | | | | | |
| Türkiye | Yemeksepeti | | | ● | ● | | | | | | | |
| | Banabi | | | ● | | | | | | | | |
| | Bitaksi | ● | | | | | | | | | | |
| | Istegelsin | | | ● | ● | | | | | | | |
| | Migros Hemen+ | | | ● | ● | ● | ● | | | | | |
| | Getir | ● | | ● | ● | ● | ● | ● | ● | | | |
| Ucrania | Raketa | | | ● | ● | | | | | | | |
| | Uber | | | | ● | ● | ● | | | | | |
| | Bolt | | | ● | ● | | | | | | | |
| | Glovo | | | ● | ● | ● | ● | | | | | |

Fuente: Compilación propia basada en las categorías de trabajo justo de Fairwork (Graham et al. 2020).
